# Real-world sex differences in healthcare utilization after cryoballoon ablation: 2-year outcomes from the Cryo Global Registry

**DOI:** 10.3389/fcvm.2025.1549002

**Published:** 2025-06-03

**Authors:** Surinder Kaur Khelae, Kyoung Ryul Julian Chun, Christian Drephal, Fernando Scazzuso, Fred J. Kueffer, Kelly A. van Bragt, Thorsten Lawrenz, Derick Todd, Paweł Ptaszyński, Csaba Földesi

**Affiliations:** ^1^ Department of Electrophysiology, Institut Jantung, Negara—National Heart Institute, Kuala Lumpur, Malaysia; ^2^ Department of Cardiology, Cardioangiologisches Centrum Bethanien, Frankfurt, Germany; ^3^ Internal Medicine and Cardiology, Sana Klinikum Lichtenberg, Berlin, Germany; ^4^ Department of Electrophysiology, Institituo Cardiovascular Buenos Aires (ICBA), Buenos Aires, Argentina; ^5^ Cardiac Ablation Solutions, Medtronic, Inc., Minneapolis, MN, United States; ^6^ Department of Cardiology and Intensive Care Medicine, Städtische Kliniken Bielefeld gem. GmbH—Klinikum Mitte, Bielefeld, Germany; ^7^ Department of Cardiology, Liverpool Heart and Chest Hospital, Liverpool, United Kingdom; ^8^ Department of Electrophysiology, Centralny Szpital Kliniczny Uniwersytetu Medycznego w Łodzi, Łódź, Poland; ^9^ Department of Electrophysiology, Gottsegen György Országos Kardiovaszkuláris Intézet, Budapest, Hungary

**Keywords:** atrial fibrillation, catheter ablation, cryoballoon, sex, female, registry

## Abstract

**Introduction:**

Cryoballoon ablation (CBA) is a well-established treatment for atrial fibrillation (AF). However, evidence on the impact of sex on outcome is sparse. This real-world analysis aims to describe outcome after CBA in both sexes.

**Methods:**

This registry is an ongoing, global evaluation of CBA procedures in standard-of-care practice. Females undergoing CBA were compared to males at baseline and 12-, and 24-months post-ablation. Serious procedure-related adverse event rate, rate of atrial arrhythmia (AA) recurrence, repeat ablation, hospitalization, cardioversion, and quality-of-life (QoL; EQ-5D-3l) were compared.

**Results:**

Of 3,089 patients with 12-month follow-up, 1,136 (36.8%) were female; and a subset of 1,099 patients (400 female) were followed through 24 months. Females presented with different comorbidities at baseline. The complication rate was low overall in females (3.9%) and males (2.7%, *p* = 0.07). At 24-month follow-up, the rate of AA recurrence was 25.0% in females and 22.1% in males and female sex was a predictor of AA recurrence (HR adj = 1.21, *p* = 0.047) and rehospitalizations (HR adj = 1.25, *p* = 0.03) in a propensity score adjusted analysis. In addition, females stayed in the hospital longer compared to males during the index ablation procedure (47.9% with 2 or more days in hospital vs. 39.3% in males, *p* < 0.01), as well as during the first hospital stay post-ablation (78.2% with 2 or more days in hospital vs. 60.2% in males, *p* < 0.01). QoL improved from baseline to 12 months in females (0.85 ± 0.15–0.90 ± 0.13) and males (0.91 ± 0.13–0.94 ± 0.11) and remained high at 24 months.

**Discussion:**

CBA performed according to standard-of-care practice was safe in both sexes. The efficacy of CBA is marginally lower in females, but 75% of females remained free from AA recurrence at 24-months and reported a meaningful improvement in QoL post ablation.

**Systematic Review Registration:**

https://clinicaltrials.gov/ct2/show/NCT02752737, The Cryo Global Registry (NCT02752737)

## Introduction

Atrial fibrillation (AF) is the most common sustained cardiac arrhythmia, and its prevalence is increasing in adults worldwide (1, 2). The worldwide prevalence of AF ranges from equal to lower in females compared to males (3, 4) and increases over time in both sexes (1). If left untreated, AF poses an increased risk for morbidity and mortality, causing a significant burden for patients and healthcare institutions (5). Moreover, the relative risk of all-cause mortality, stroke, cardiovascular mortality, cardiac events, and heart failure in individuals with AF vs. those without AF is higher in females compared to males (6). Catheter ablation (CA) is a well-adopted evidence-based safe and efficacious treatment for managing AF and preventing recurrences of AF (2, 7). Despite the increased cardiovascular risks, females are less likely to be referred to a specialized outpatient arrhythmia clinic for management of AF, and females are less likely to undergo electrical cardioversion or catheter ablation (8). In addition, females are large underrepresented in large randomized controlled trials (9, 10). Several catheter ablation studies have shown sex-based differences in atrial arrhythmia recurrences (11–13). Differences in co-morbidity burden, as well as the underlying electrophysiological substrate may explain the lower arrhythmia-free survival in females. Studies have shown lower mean voltage, significant conduction slowing, more fractionated electrograms and more fibrosis across the atria of females with AF, as well as decreased left atrium (LA) strain and higher stiffness, compared to males (14–17). Literature on sex-specific cryoballoon ablation (CBA) outcomes is limited. Some studies have shown that females with AF may be more symptomatic and report a lower quality of life, although it is unclear whether this is related to sex differences (5). The recent 2024 expert consensus statement indicates that sex disparity in AF outcomes may be attributed to factors such as delayed catheter ablation referral, differences in anatomical features, and differences in burden of associated comorbidities (7). This sub-analysis of the Cryo Global Registry aims to evaluate the real-world experience of CBA in a large cohort of males and females, comparing their baseline characteristics, safety and efficacy outcomes, disease symptom burden, and health care utilization after propensity score matching. A better understanding of routine CBA in females with AF can lead to improved quality of care and tailored treatment in this underrepresented patient population.

## Materials and methods

### Study design

The Cryo Global Registry (NCT02752737) is an ongoing, prospective, multicenter post- market registry to evaluate the outcomes of CBA procedures performed with the Arctic Front Advance Family of cryoablation catheters (Medtronic, Inc.) for the treatment of AF. A global steering committee of international physicians oversees data quality, statistical analyses, and publication milestones. Data collection was in alignment with the principles outlined in the Declaration of Helsinki (2013) and Good Clinical Practices. Patients provided written informed consent prior to any study specific procedures, and the study was approved by local institutional review boards and ethics committees at each participating center. All procedures performed in the registry were performed according to standard-of-care hospital protocols at the time of data collection. More details on the study design and the cryoballoon procedure have been previously described (18, 19).

### Patient population

Patients ≥18 years old with a planned CBA procedure were eligible for inclusion in the registry and were not excluded based on pre-existing medical conditions nor baseline characteristics. This analysis included patients with a minimum of 12 months of follow-up post ablation. Patients with prior cardiac ablations and longstanding persistent AF patients (continuous AF >12 months) were excluded. Real-world experience of CBA between sexes was analyzed in patients enrolled between May 2016 and July 2020 at 96 international centers in 37 countries. Classification of AF was done in accordance with international guidelines; paroxysmal AF (PAF; AF that terminates spontaneously or with intervention within 7 days of onset) or persistent AF (PsAF; continuous AF that is sustained beyond 7 days and ≤12 months) (7).

### Cryoballoon ablation procedure

The cryoballoon ablation procedure was conducted according to standard of care at each participating site. This procedure has been described in detail in previous publications (18, 20–22). The cryoballoon ablation procedure was conducted according to standard of care at each participating site. This procedure has been described in detail in previous publications (citations included in the manuscript). In brief, after patient sedation, the LA was accessed through a transseptal puncture. A 15-F OD steerable sheath was used to introduce a 23 or 28 mm cryoballoon ablation catheter (Medtronic, Inc) into the LA. The cryoballoon catheter and sheath were maneuvered to the targeted pulmonary vein (PV) with either a J-tip guidewire or a inner-lumen octopolar/decapolar circular mapping catheter (Medtronic, Inc). After antral occlusion of the targeted PV was achieved, the cryoapplication was triggered. The number and duration of cryoapplications per PV were determined by the operating physician. Sites were recommended to monitor phrenic nerve function during right-sided PVI by pacing with a diagnostic catheter. All cryoapplications were terminated upon detection of an attenuated diaphragmatic response. Application of additional lesions (other non-PVI ablation), adjunctive imaging (e.g., intracardiac echocardiography, three-dimensional electroanatomical mapping), intraprocedural esophageal temperature monitoring and use of ablation tools were determined by the operating physician for each patient. PVI was confirmed by demonstration of entrance and/or exit block following the ablation. Pre-procedure, intra-procedure, and post-procedure tools and techniques to guide, monitor and assess the CBA procedure were all applied at the discretion of the physician per patient. Physicians also determined appropriate peri-procedural anticoagulation, initiation or continuation of AADs, and followed local standard-of-care policies to discharge patients from the hospital.

### Patient follow-up and endpoints

Patients were followed through 12 or 24 months according to the hospital's standard-of care. The study protocol required annual status visits; however, the method and frequency of rhythm monitoring was not dictated by the study protocol. Monitoring methods included, but were not limited to, 12-lead electrocardiogram, Holter monitor, trans-telephonic monitor, insertable cardiac monitor, pacemaker, and/or implantable cardioverter defibrillator.

Efficacy and safety after the index-CBA procedure throughout 24 months of follow-up were reported. Efficacy was assessed by freedom from ≥30 s recurrence and atrial arrhythmias [AA; defined as AF/atrial flutter (AFL)/atrial tachycardia (AT)] between a 90-day blanking period and 24-month follow-up. During the 90-day blanking period, recurrent atrial arrhythmias could be managed with cardioversion, repeat ablation, and medication per hospital standard of care practices. All reported adverse events from Cryo Global registry were assessed for seriousness/relatedness by the investigator and by safety team. Adverse events were classified by the treating physician by seriousness and relatedness to the procedure or CBA system. Serious adverse events included all events that led to a) death, or b) to a serious deterioration in health that resulted in either (1) a life-threatening illness or injury, (2) a permanent impairment in body structure or function, (3) in-patient or prolonged hospitalization, or (4) medical intervention to prevent life-threatening illness or injury.

Patient demographics and procedural characteristics were assessed. Predefined AF-related symptoms and quality-of-life (QoL, measured by EQ-5D-3l) were reported by the patient at baseline, 12, and 24 months. The EQ-5D-3l questionnaire measures five dimensions of health, including: (1) mobility, (2) selfcare, (3) physical activities, (4) pain and discomfort, and (5) anxiety and depression. Each question has three levels of response indicating no problem, some problem, or extreme problem. Rate of repeat ablations and cardioversions (in addition to all-cause non-procedure related hospitalizations post-index procedure) were reported throughout 24 months of follow-up.

### Statistical analysis

Baseline characteristics and clinical data were summarized using the appropriate summary statistics; continuous variables are summarized as mean and standard deviation, and categorical variables are summarized as counts and percentages. Differences in baseline characteristics between the male and female cohorts were tested with a two-sample *t*-test for continuous variables and Fisher's exact test for categorical variables. Similarly, differences in procedural characteristics were tested with a two-sample *t*-test for continuous variables and Fisher's exact test for categorical variables. For skewed continuous data, Wilcoxon rank-sum test was utilized. Kaplan–Meier methods were used to estimate the 24-month freedom from arrhythmia recurrence, repeat ablations, cardioversions and hospitalizations. Standard error was approximated with Greenwood's formula. Unadjusted and adjusted hazard ratios were calculated with Cox regression. Unadjusted models included only group cohort (female vs. male) as a covariate in the model. Adjusted models accounted for differences in baseline characteristics between the female and male cohorts utilizing propensity score methods, specifically propensity score covariate adjustment method. In the adjusted Cox model, group cohort (female vs. male) and propensity score were included as covariates with propensity score calculated as follows. Logistic regression was used to calculate propensity score for each subject where group cohort (female vs. male) was the dependent variable, and baseline variables from Table 1 including age, body mass index, type of AF (paroxysmal vs. persistent), years diagnosed with AF, history of atrial flutter, history of atrial tachycardia, left atrial diameter, left ventricular ejection fraction, number of prior failed AADs, hypertension, baseline NYHA, prior myocardial infarction, prior stroke/TIA, coronary artery disease, diabetes and sleep apnea were included as covariates. CHA_2_DS_2_-VASc score was excluded as it is a composite of other variables in the model (heart failure, hypertension, age, diabetes, prior stroke/transient ischemic attack).

**Table 1:** Baseline characteristics (female vs. male).

Patient Characteristics	Female	Male	*P*-value^e^
	(*N* = 1,136)	(*N* = 1,953)	
Age (years)	64 ± 10	59 ± 12	<0.01
Body mass index (kg/m^2^)	27 ± 5	28 ± 5	0.40
CHA_2_DS_2_-VASc Score	2.9 ± 1.4	1.4 ± 1.3	<0.01
CHA_2_DS_2_-VA score	1.9 ± 1.4	1.4 ± 1.3	<0.01
Paroxysmal AF	959 (84.4%)	1,562 (80.0%)	<0.01
Years diagnosed with AF^a^			
Mean ± SD	2.9 ± 4.1	3.3 ± 5.0	0.40^f^
Median [IQR]	1.4 [0.4–3.8]	1.4 [0.4–4.1]	
History of atrial flutter	66 (5.8%)	141 (7.2%)	0.14
History of atrial tachycardia	25 (2.2%)	26 (1.3%)	0.08
Left atrial diameter^b^ (mm)	40 ± 7	41 ± 7	<0.01
Left atrial diameter indexed to body size (LAD/BMI)	1.50 ± 0.32	1.54 ± 0.28	<0.01
Left ventricular ejection fraction^c^ (%)	61 ± 9	59 ± 9	<0.01
Number of failed AADs	0.8 ± 0.8	0.7 ± 0.7	0.14
Hypertension	685 (60.3%)	1,005 (51.5%)	<0.01
Baseline NYHA^d^ Class ≥ II	178 (17.3%)	186 (10.5%)	<0.01
Prior myocardial Infarction	19 (1.7%)	64 (3.3%)	<0.01
Prior stroke/transient ischemic attack	67 (5.9%)	118 (6.0%)	0.94
Coronary artery Disease	88 (7.7%)	240 (12.3%)	<0.01
Diabetes	138 (12.1%)	267 (13.7%)	0.25
Sleep apnea	40 (3.5%)	95 (4.9%)	0.08

Abbreviations: AF, atrial fibrillation; STD, standard deviation; AAD, antiarrhythmic drug, NYHA, New York Heart Association.

^a^ AF Diagnosis date reported in 1,061 females and 1,822 males. Median [IQR] also reported for years diagnosed with AF due to data being skewed.

^b^ Left atrial diameter reported in 767 females and 1,250 males.

^c^ Left ventricular ejection fraction reported in 952 females and 1,621 males.

^d^ NYHA assessed at baseline in 1,031 females and 1,772 males.

^e^ two-sample *t*-test for continuous variables and exact test for categorical variables.

^f^ Wilcoxon rank-sum test due to data are skewed.

Multiple imputation methods were utilized in the propensity score modeling, imputing baseline data for patients missing data. Multivariate imputation by fully conditional specification methods were utilized with logistic regression method specified for classification variables and regression methods utilized for continuous variables (23). Differences in safety event rates between male and female patients were assessed with a Fisher's exact test. Differences in first hospitalization post-ablation length of stay was assessed with Wilcoxon rank-sum test. Changes in symptoms and QoL from baseline to 24-months were assessed with a *t*-test. Values of *P* < 0.05 were considered significant. Statistical analyses were conducted using SAS software version 9.4 (SAS Institute, Cary, North Carolina).

## Results

### Patient demographics and follow-up

A total of 3,089 patients (36.8% female) that were enrolled and treated in the Cryo Global Registry had 12-month follow-up data available, and a subset of 1,099 patients had 24-month data available. Attrition was low with 301 (9.7%) of patients exiting early, including: 53 withdrawn by the investigator early, 188 lost to follow-up, 49 patients requested withdrawal, and 11 for “other” reasons. On average, patients had a follow-up visit 2.3 ± 1.6 times during the first 12 months post CBA, and 1.2 ± 0.9 times in the second year of follow-up, per the hospital's standard-of-care. There was no difference in the mean number of follow-up visits in females and males through 12 months (2.3 ± 1.6 vs. 2.3 ± 1.6, *p* = 0.47), nor during the 12-to-24-month follow-up period (1.2 ± 0.8 vs. 1.2 ± 0.9, *p* = 0.73). In total, 23 (0.7%) deaths were reported during the follow-up period, of which three occurred within 30 days of the procedure. Of the three deaths, one was confirmed to be related to the procedure (cerebrovascular accident), and the other two deaths were due to infective exacerbation of chronic obstructive airways disease and acute myocardial infarction, respectively.

Baseline characteristics are presented in Table 1. Females were on average older (64 ± 10 vs. 59 ± 12, *p* < 0.01) than male study patients and presented more often with PAF (84.4% vs. 80.0%, *p* < 0.01). In addition, females had a significantly higher CHA_2_DS_2__−_VASc score (2.9 ± 1.4 vs. 1.4 ± 1.3, *p* < 0.01) as well as a higher CHA_2_DS_2__−_VA score (excluding the female sex component; 1.9 ± 1.4 vs. 1.4 ± 1.3; *p* < 0.01). Furthermore, females presented more often with hypertension (60.3% vs. 51.5%, *p* < 0.01), but they less often presented with prior myocardial infarction (1.7% vs. 3.3%, *p* < 0.01) and history of coronary artery disease (7.7% vs. 12.3%, *p* < 0.01) compared to males. Females had a significantly higher left ventricular ejection fraction (61 ± 9 vs. 59 ± 9, *p* < 0.01). In addition, the left atrial (LA) diameter (40 ± 7 vs. 41 ± 7, *p* < 0.01) and LA diameter corrected for BMI (1.50 ± 0.32 vs. 1.54 ± 0.28, *p* < 0.01) were smaller in females compared to males, respectively. There was no difference in the number failed antiarrhythmic drugs (AADs) at baseline.

### Procedural characteristics

Procedure times, presented in Table 2, were not statistically different between sexes. A trend was observed towards more additional ablations other than pulmonary vein isolation (PVI) and cavotricuspid isthmus ablation (CTI) in females vs. males (6.7% vs. 5.0%, *p* = 0.06). Length of hospital stay during the index procedure was longer for females (2.24 ± 6.02 days vs. 2.17 ± 12.95 days in males, *p* < 0.01). More females reported length of stay of 2 or more days (47.9% vs. 39.3% of males, *p* < 0.01). At discharge, 580 (51.1%) females and 1,013 males (51.9%) males were on AADs. At 12 months, 274 (26.2%) females and 432 (24.7%) males were on AADs. At 24 months, 118 (31.8%) females and 198 (31.4%) males were on AADs.

**Table 2:** Procedural characteristics.

Procedure characteristics	Female	Male	*P*-value^a^
	(*N* = 1,136)	(*N* = 1,953)	
Total Procedure Time (min)	82 ± 35	82 ± 34	0.69
Left Atrial Dwell Time (min)	54 ± 24	54 ± 25	0.61
Total Cryo Fluoroscopy time (min)	18 ± 16	18 ± 23	0.80
Total cryoapplication time (min)^b^	19 ± 7	19 ± 7	0.99
Number of applications per Vein^c^	1.6 ± 0.9	1.6 ± 0.9	0.33
Duration of Cryoapplication (sec)^d^	182 ± 51	183 ± 54	0.42
Sedation method			0.07
General anesthesia	420 (37.0%)	786 (40.2%)	
Conscious sedation	716 (63.0%)	1,166 (59.7%)	
Pre-procedural imaging (CT and/or MRI)	209 (18.4%)	403 (20.7%)	0.13
3D Electroanatomical Mapping (CARTO, NAVx)	167 (14.7%)	275 (14.1%)	0.63
Intracardiac echocardiography	283 (24.9%)	460 (23.6%)	0.41
Phrenic nerve monitoring	1,127 (99.2%)	1,935 (99.1%)	0.84
Esophageal temperature monitored	488 (43.0%)	796 (40.8%)	0.24
PVI acute success	1,079 (95.0%)	1,868 (95.6%)	0.42
PVI touch-up with Focal Cryo catheter	2 (0.2%)	2 (0.1%)	0.63
PVI touch-up with Focal RF Catheter	13 (1.1%)	29 (1.5%)	0.52
Additional ablation lesions			
CTI line	119 (10.5%)	240 (12.3%)	0.13
Other non-PVI ablation	76 (6.7%)	98 (5.0%)	0.06
Length of index hospital stay (Days)			<0.01
Same day discharge	37 (3.3%)	134 (6.9%)	
1 day	554 (48.8%)	1,051 (53.8%)	
2 or more days	544 (47.9%)	515 (39.3%)	

Abbreviations: CT, computerized tomography; MRI, magnetic resonance imaging; PVI, pulmonary vein isolation; RF, radiofrequency; CTI, cavo-tricuspid isthmus.

^a^ two-sample *t*-test for continuous variables and exact test for categorical variables.

^b^ Cryoablation energy application data reported in 1,132 females and 1,951 males.

^c^ 4,453 pulmonary veins treated in 1,132 females, 7,732 pulmonary veins treated in 1,951 males.

^d^ 7,053 total cryoapplications in females, 12,116 total cryoapplications in males.

### Efficacy and safety

**Figure 1:**
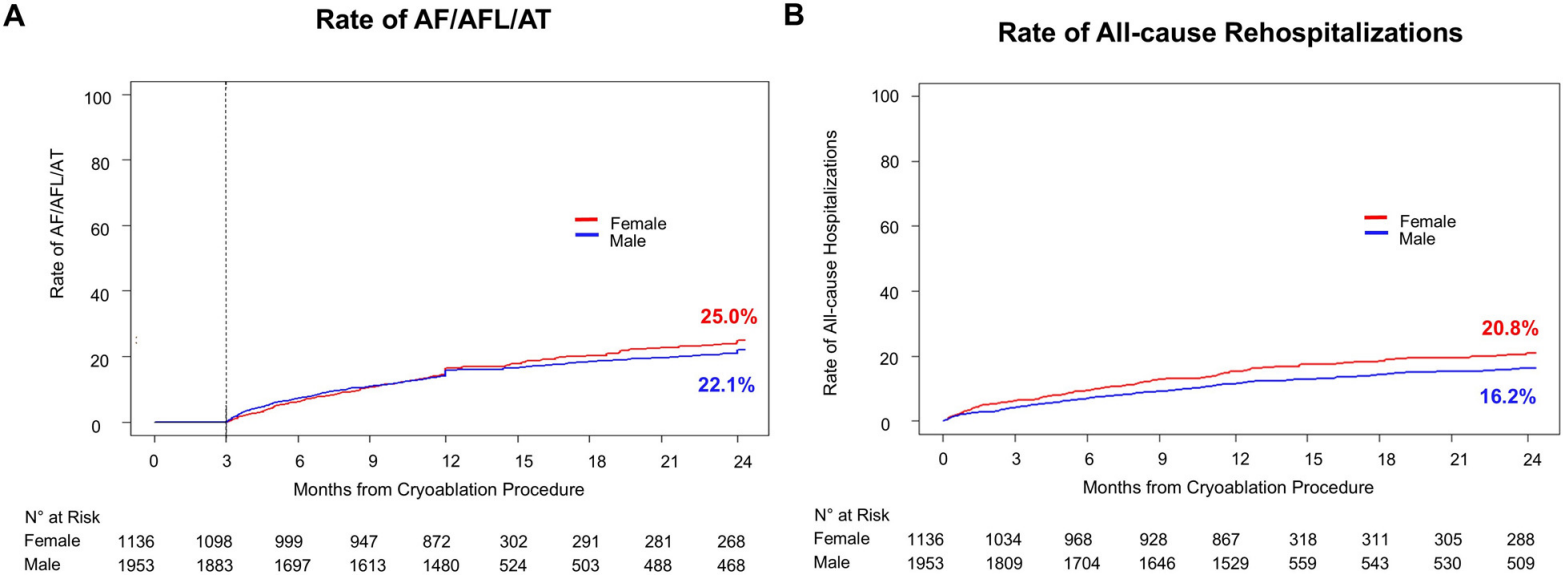
Rate of atrial arrhythmia recurrence and All-cause rehospitalizations over 24-months. Kaplan–Meier graphs of **(A)** the rate of a ≥30 s recurrence of atrial fibrillation (AF)/atrial flutter (AFL)/atrial tachycardia (AT) after the blanking period through 24 months of follow-up and **(B)** all-cause rehospitalizations through 24 months of follow-up. Females are represented in red lines, males in blue lines.

Long-term efficacy is depicted in Figure 1A. The rate of AA recurrence was 25.0% in females and 22.1% in males at 24-months follow-up. Female sex was a predictor of AF/AFL/AT recurrence rate (HR adj = 1.21, *p* = 0.047) in a propensity score adjusted analysis (Table 3). Although not statistically significant, repeat ablations and cardioversion were also higher in females with a 15% increase in repeat ablation risk (HR adj = 1.15, *p* = 0.35) and 28% increase in cardioversion risk (HR adj = 1.28, *p* = 0.21). Arrhythmia monitoring was conducted per standard of care at study centers. ECG and Holter monitoring rates were similar between male and female. In the first 12-months, female patients received 1.6 ± 1.7 12-lead ECGs on average and 1.6 ± 1.7 for males (*p* = 0.65). Holter rates in the first 12-months were 0.8 ± 1.1 in female and 0.8 ± 1.1 in male patients (*p* = 0.78). In the 12–24 month time period, ECG and Holter rates were also similar (ECG: 0.7 ± 1.0 in females, 0.7 ± 1.0 in males, *p* = 0.45; Holter: 0.3 ± 0.6 in females, 0.3 ± 0.5 in males, *p* = 0.15). Intracardiac loop recording was available in 2.1% of patients (2.3% female, 1.9% male).

**Table 3:** Cox regression analysis of efficacy, repeat ablation, cardioversion and all-cause hospitalization.

24-month outcome	Female (*n* = 1,136)	Males (*n* = 1,953)	Hazard ratio (unadjusted)^a^	Hazard ratio (adjusted)^b^
Recurrence of AF/AT/AFL	25.0% (95% CI: 21.7–28.7%)	22.1% (95% CI: 19.8–24.7%)	1.08 (95% CI: 0.91–1.29)	1.21 (95% CI: 1.002–1.46)
			*p* = 0.38	*p* = 0.047
Repeat ablation	10.7% (95% CI: 8.5–13.5%)	9.9% (95% CI: 8.2–11.9%)	1.05 (95% CI: 0.80–1.37)	1.15 (95% CI: 0.85–1.54)
			*p* = 0.75	*p* = 0.35
Cardioversion	6.0% (95% CI: 4.4 −8.1%)	4.1% (95% CI: 3.2%–5.4%)	1.34 (95% CI: 0.93–1.94)	1.28 (95% CI: 0.87–1.92)
			*p* = 0.12	*p* = 0.21
Rate of all-cause rehospitalization	20.8% (95% CI: 17.9–24.0%)	16.2% (95% CI: 14.2–18.4%)	1.35 (95% CI: 1.11–1.63)	1.25 (95% CI: 1.02–1.53)
			*p* < 0.01	*p* = 0.03
Length of stay during first rehospitalization post ablation (days)				*p* < 0.01^c^
• Mean ± SD	5.7 ± 7.7	3.5 ± 5.6		
• Median [IQR]	3 [2–6]	2 [1–4]		
• Same day discharge	13 (6.7%)	36 (14.5%)		
• 1 day stay	29 (15.0%)	63 (25.3%)		
• ≥2 days stay	151 (78.2%)	150 (60.2%)		
Number of times rehospitalized post ablation				
• 0	943 (83.0%)	1,703 (87.2%)		
• 1	146 (12.9%)	198 (10.1%)		
• ≥2	47 (4.1%)	52 (2.7%)		

Abbreviations: AF/AT/AFL, atrial fibrillation/atrial tachycardia/atrial flutter; SD, standard deviation; IQR, inter-quartile range.

^a^ Cox regression.

^b^ Multivariable Cox regression with propensity score as covariate. Baseline variables from Table 1 included as covariates in propensity score modeling to account for differences observed in patient characteristics between males and females.

^c^ Wilcoxon rank-sum test.

The rate of serious procedure-related adverse events was low overall in females (3.9%) and males (2.7%, *p* = 0.07). A detailed list of adverse events per sex is provided in Table 4. Of particular note, the number of phrenic nerve injuries classified as serious by the investigators was low and similar in both cohorts (0.8% in females vs. 0.3% in males, *p* = 0.10). Also, similar rates of cardiac tamponade, cardiac perforation, or pericardial effusion were reported, with 7 (0.6%) reports in females and 5 (0.3%, *p* = 0.14) in males.

**Table 4:** Serious procedure-related adverse events.

Serious Procedure-Related Complications	Female	Male	*P*-value^g^
	(*N* = 1,136)	(*N* = 1,953)	
Total	51 (44, 3.9)	55 (52, 2.7)	0.07
Atrial septal defect	0 (0, 0.0)	1 (1, 0.1)	
Cardiac failure	1 (1, 0.1)	1 (1, 0.1)	
Cardiac tamponade, perforation, pericardial effusion	7 (7, 0.6)	5 (5, 0.3)	
Femoral artery aneurysm	1 (1, 0.1)	0 (0, 0.0)	
Fluid overload	0 (0, 0.0)	1 (1, 0.1)	
Groin-site complication^a^	12 (10, 0.9)	14 (14,0.7)	
Myocardial infarction or ischemic cardiac event^b^	3 (3, 0.3)	2 (2, 0.1)	
Pericarditis	2 (2, 0.2)	2 (2, 0.1)	
Phrenic nerve injury	9 (9, 0.8)	6 (6, 0.3)	
Postoperative hypotension	1 (1, 0.1)	3 (3, 0.2)	
Presyncope	0 (0, 0.0)	2 (2, 0.1)	
Pulmonary or bronchial complication^c^	5 (5, 0.4)	4 (4, 0.2)	
Sepsis	1 (1, 0.1)	0 (0, 0.0)	
Stress cardiomyopathy	1 (1, 0.1)	0 (0, 0.0)	
Stroke or TIA of any cause^d^	2 (2, 0.2)	3 (3, 0.2)	
Supraventricular arrhythmias^e^	4 (4, 0.4)	7 (7, 0.4)	
Urinary retention	0 (0, 0.0)	1 (1, 0.1)	
Other^f^	2 (2, 0.2)	3 (3, 0.3)	

Abbreviations: TIA, transient ischemic attack.

Numbers are presented as Number of Events (Number Patients, % Patients).

^a^ Arteriovenous fistula, arteriovenous fistula aneurysm, arteriovenous fistula site hematoma, femoral artery dissection, hematoma, incision site hematoma, puncture site hematoma, vascular access site hemorrhage, vascular pseudoaneurysm, vascular pseudoaneurysm ruptured, vessel puncture site discharge, vessel puncture site hematoma.

^b^ Angina pectoris, coronary arteriospasm, myocardial infarction.

^c^ Hematemesis, hemoptysis, hypercapnia, pneumothorax, pulmonary embolism, pneumonia, pleurisy.

^d^ Cerebral infarction, cerebrovascular accident, ischemic stroke, lacunar stroke.

^e^ Atrial fibrillation, atrial tachycardia, nodal arrhythmia, sinus bradycardia.

^f^ Pyrexia, Lip Injury, Face Injury, Headache.

^g^ Fisher's exact test.

### Rehospitalizations

Females had a 25% increase in the risk for rehospitalization post ablation (HR adj = 1.25, *p* = 0.03) after a propensity adjusted analysis (Figure 1B). In addition, females stayed in the hospital longer (Table 3) compared to males (5.7 ± 7.7 days vs. 3.5 ± 5.6, respectively; *p* < 0.01) and 78.2% of females stayed in the hospital ≥2 days vs. 60.2% of males, *p* < 0.01. The majority of patients was not rehospitalized (83.0% of females and 87.2% of males) in the 24 months post ablation. However, if hospitalized, this happened more often in females than males (4.1% females with ≥ 2 hospitalizations vs. 2.7% males, *p* < 0.01). The most common reason for the first report of an all-cause rehospitalization was AA recurrence in 124 (10.9%) female and 174 (8.9%) male patients. No other reason for the first report of an all-cause rehospitalization was prominent in either cohort.

### Symptom burden and quality-of-life

**Figure 2:**
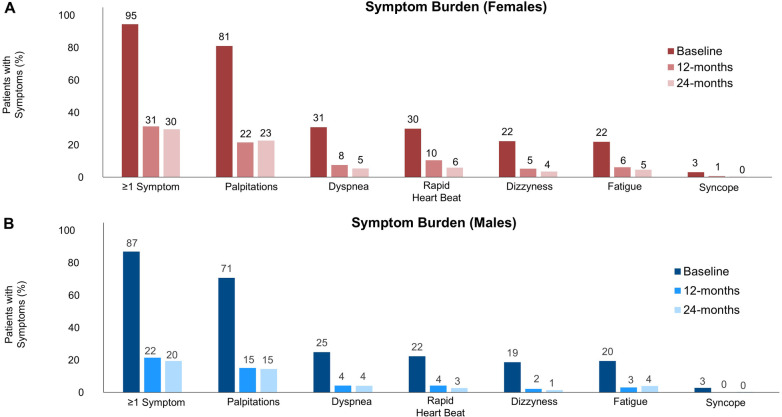
AF-related symptom burden over 24-months. Proportion of **(A)** female and **(B)** male patients with predefined symptoms (dizziness, palpitations, rapid heartbeat, dyspnea, fatigue, syncope) related to AF at baseline, 12-month, and 24-month follow-up.

The percentage of patients reporting each AF-related symptom per sex can be found in Figure 2. Females had more AF-related symptoms at baseline than males (1.9 ± 1.2 vs. 1.6 ± 1.1, *p* < 0.01). Females reported a similar reduction of symptoms [−1.38 (95% CI:−1.46, −1.29) symptoms] from baseline to 12 months compared to males [−1.30 (95% CI: −1.36, −1.25) symptoms, *p* = 0.13].

QoL as measured by the EQ-5D-3l summary score and VAS analog scale are summarized in Table 5. Females had a significantly lower QoL score at baseline compared to males (EQ-5D: 0.85 ± 0.15 vs. 0.91 ± 0.13, respectively, *p* < 0.01; VAS analog scale: 70.3 ± 17.0 vs. 75.3 ± 16.2, respectively, *p* < 0.01). Both sexes experienced a statistically significant improvement in QoL from baseline to 12-months (*p* < 0.01 for both sexes) that remained high at 24-months. The absolute improvement in EQ-5D summary score from baseline to 12-months was higher in females compared to males (0.05 vs. 0.03 points, respectively, *p* < 0.01). The VAS analog scale also improved significantly more in females compared to males (8.1 vs. 6.6 points, respectively, *p* = 0.03).

**Table 5:** Quality of life.

A) EQ-5D-3L Summary Score								
Sex	Baseline		Month 12				Month 24	
	N	EQ-5D score	N	EQ-5D score	Difference from baseline	*p*-value	N	EQ-5D score
Female	998	0.85 ± 0.15	998	0.90 ± 0.13,	0.051	<0.01	349	0.90 ± 0.14
				*p* < 0.01^b^	(95% CI: 0.042–0.061)			
Male	1,675	0.91 ± 0.13,	1,675	0.94 ± 0.11,	0.034		580	0.95 ± 0.10
		*p* < 0.01^a^		*p* < 0.01^c^	(95% CI: 0.028–0.040)			

B) VAS Analog Score								
Sex	Baseline		Month 12				Month 24	
	N	VAS score	N	VAS score	Difference from baseline	*p*-value^g^	N	VAS score
Female	995	70.3 ± 17.0	995	78.4 ± 15.6 *p* < 0.01^e^	8.1	0.03	349	77.2 ± 14.9
					(95% CI: 7.0–9.2)			
Male	1,673	75.3 ± 16.2,	1,673	81.9 ± 13.7 *p* < 0.01^f^	6.6		580	80.7 ± 13.5
		*p* < 0.01^d^			(95% CI: 5.8–7.4)			

^a^ Wilcoxon rank sum test comparing male vs. female baseline EQ-5D.

^b^ *t*-test. Change in EQ-5D from baseline to 12-months within female subgroup.

^c^ *t*-test. Change in EQ-5D from baseline to 12-months within male subgroup.

^d^ *t*-test comparing male vs. female baseline VAS.

^e^ *t*-test. Change in VAS from baseline to 12-months within female subgroup.

^f^ *t*-test. Change in VAS from baseline to 12-months within male subgroup.

^g^ two sample *t*-test, change from baseline to 12-months compared between males and females.

## Discussion

### Primary findings

In this real-world observation, females made up 37% of the total cohort. Females undergoing CBA for AF had significant differences in baseline characteristics compared to males. There were no differences in procedure times, but females stayed in the hospital longer following index-CBA. In addition, there was no significant difference between females and males in complication rate, repeat ablations, and cardioversions. However, female sex was an independent predictor of AA recurrences and rehospitalizations at 24 months of follow-up. Females had a higher symptom burden and lower QoL compared to males at baseline. Both sexes reported a significant improvement in symptoms and QoL at 12-months that remained high at 24-months, and the absolute improvement in QoL after 12-months was higher in females.

### Patient population

The percentage of females enrolled in previous CBA studies ranged from 25% to 32%. (12, 24, 25) Importantly, the percentage of females in this registry is higher (37%); however, this percentage of female participation is still lower than the reported median percentage of females with AF in the total population (45.3%, IQ 40.6%-47.2%) (10). These data indicate that a “rhythm control treatment gap” may still exist for females with AF. It has been suggested that the presence of symptoms atypical to AF in females may delay diagnosis and therapy (3). A recent single-center study has shown that females were not more reluctant to accept CA when offered (26), and reasons for this disparity warrant further investigation.

### Clinical outcomes

Several studies and meta-analyses have compared outcomes in females and males after index CA, with contradictory results (8, 11–13, 24, 25, 27–36). However, only a limited number of observational studies have investigated the impact of sex on differences in outcome following a CBA procedure, specifically (12, 24, 25). Two observational studies showed higher rates of adverse events in females undergoing CBA (12, 24, 25). Ricciardi, et al. reported similar complication rates between sexes (12). Common complications in females were phrenic nerve injury, cardiac tamponade, cardiac perforation, and pericardial effusion (12, 24, 25). In the current analysis, overall serious procedure-related adverse event rate was similarly low in both sexes and there was no difference in the rate of phrenic nerve injury, and cardiac tamponade, cardiac perforation, and pericardial effusion. The low adverse event rate found here is in alignment with other contemporary CBA trials (22, 37).

Previous CBA publications have reported differences in efficacy results between sexes. A recent publication from Ekrami et al. (25) showed no statistical difference in the rate of AA recurrences between sexes at 12 months. Looking at longer follow-up durations, Hermida et al. (12) showed that females with PAF undergoing cryo-PVI had a higher risk of AA recurrence after 24 and 36 months of follow-up. In the 1-STOP Italian registry (24), the 24-month freedom from AF was not found to be statistically different between sexes, but a trend was observed for a lower AF-free survival in females. In the current large real-world analysis, females also had an increased risk of AA recurrences after 24 months of follow-up, after correcting for differences in baseline characteristics. The majority of females still benefited from CA as 75% of females remained free from AA recurrence at 24-months. Literature suggests that females often present with more advanced structural remodeling at the time of ablation and a lower incidence of rotational activity outside the pulmonary veins (14, 15). These mechanistic findings may potentially account for the post-ablation differences and higher AA recurrences in females (15).

In the present analysis, females also had a significantly longer hospital stay at index-CBA and first rehospitalization, as well as more rehospitalizations post-ablation compared to males. AA recurrence was the main reason for rehospitalization, but it did not explain all of the difference in rehospitalization rate between sexes. Although minimal, some other causes of rehospitalizations include sinus node dysfunction [5(0.4%) females, 3(0.2%) males], diverticulitis [3(0.3%) females, none in male], pneumonia [2(0.2%) females, 3(0.2%) males], and hypertension [2(0.2%) females, 1(0.1%) male]. Index-hospital stay is known to be longer for females (13, 32), and physician precaution (for an anticipated increased risk for cardiovascular complications in females undergoing CA) may be one potential reason for this observation (32). Although females in this study reported more symptoms and had an increased risk of recurrences; they did not undergo more additional treatment for AAs (meaning repeat ablations or cardioversions) than males within 24 months of follow-up. Female sex was also reported to be associated with a 36% increased risk of cardiovascular rehospitalization compared to males in a retrospective analysis of the FIRE and ICE randomized controlled trial (13). A predefined sub-analysis of the EAST-AFNET4 trial showed that early rhythm intervention led to improved cardiovascular outcome and hospitalization for heart failure or acute coronary syndrome in both sexes (despite females having higher CHA_2_DS_2_-VASc) compared to usual care, which underlines the importance of early treatment in females with AF (38). Further studies are needed to better understand the differences in hospitalization between sexes.

The current findings on sex differences in QoL and symptoms confirm earlier results in literature; females report a lower QoL (26, 32, 39) and higher symptom burden (12, 24, 26, 39) at baseline compared to males, regardless of the QoL questionnaire or symptoms score used, and CA results in an absolute improvement of QoL and symptoms in females with PAF and PsAF that is similar (32–34) or better (26) compared to males. The absolute improvement in QoL in the current analysis (females: 0.05, males: 0.03) was within the range of the minimally clinically important difference (MCID: 0.03–0.52) (40) reported in literature for the EQ-5D QoL instrument.

In summary, this study should encourage physicians to evaluate females for non-traditional AF disease symptoms and consider earlier rhythm management treatment from the time of diagnosis in line with current AF guidelines (2, 5). Also, female patients should not delay general cardiac care management, and they should proactively seek out specialized cardiac care, including electrophysiological intervention. These results may also apply to emerging Pulsed Field Ablation (PFA) technologies. Regardless of the energy type, PVI remains the cornerstone of AF ablation (2, 5, 7). Real-world sex-based outcomes of PFA are being collected in large registries (e.g., MANIFEST, 1-STOP Italian Registry) that will provide insights in the near term.

### Limitations

It is acknowledged that there are several limitations because of the observational study design. The analysis describes real-world usage and outcomes of CBA. Patients were not excluded based on pre-existing baseline characteristics, and females enrolled in the Cryo Global Registry had more comorbidities than males. Statistical methods were utilized to account for these differences but are limited by the data collected in this study. However, there may be other confounders (e.g., lifestyle factors, socioeconomic status and center-specific procedural protocols, and medication usage) that could not be accounted for. Furthermore, the follow-up was conducted per the hospital's standard-of-care, and consequently, only a subset of patients with 12-month follow-up data were followed up for 24 months. Lastly, site-specific arrhythmia monitoring methods were not uniform across centers. Hence, it is possible that asymptomatic arrhythmias could have been under reported.

## Conclusion

CBA performed according to standard-of-care practice was safe in both sexes. Although the benefit of CA was marginally lower in females compared to males, the majority of females remained free from AA recurrence at 24-months and reported a meaningful improvement in QoL and symptom burden at 24 months post ablation. This report may help to close the “rhythm control treatment gap” and improve access to CBA therapy for females suffering from AF.

## Data Availability

The datasets presented in this article are not readily available because of patient privacy and informed consents, including the potential for release of protected health information and adherence to global country standards on privacy. Requests to access the datasets should be directed to Medtronic, kelly.van.bragt@medtronic.com.
